# On Medicinal Saturation

**Published:** 1824-04

**Authors:** 


### Art. IV.—*On Medicinal Saturation.* By Dr. Kinglake.

Medicinal saturation consists in so far filling the system with the peculiar influence of the article that may be employed, as not to admit of its being further extended without incurring injurious effects. A great variety of effect may be produced before this point be fully reached. The excitability of the different structures of the animal frame is such as to be susceptible of various impressions from medicinal agents, without saturating the system with their agency. The sympathy subsisting between kindred textures induces a speedy participation or diffusion of medicinal agency, that rapidly dissipates its influence, and prevents its being slowly disseminated and established in the actions of life. Were it not for this protecting property in vital power, accidental impressions would be incessantly changing and undermining the natural conditions of living and healthy action. It is by the natural security afforded by the healthful state, that an exemption is furnished from the saturating and hurtful influence of powerful agents.

The point of saturation to which certain medicines may attain cannot in any case be effected, but by superinducing on the actions of life the peculiar influence which such agents may be capable of exerting. Vital action, generally considered, results from an appropriate variety of power, so associated and combined as to produce a definite effect. If this natural process be disturbed, disease would ensue, requiring to be remedied by such counteracting aid as may restore the healthful state of vital action. It is the legitimate object of all medicinal agents to subdue and adjust morbid derangement of vital power. In attempting this beneficial purpose, adequate remedies must be employed. They must be sufficiently powerful to overcome the diseased action against which they are directed. This can only be effected by inducing such changes in the action of vital power as may re-instate its healthful condition. Before this can be accomplished, it is often necessary to persist in the use of powerful medicines, until an influence has been produced that temporarily supersedes the general character of vital action, afforded by the ordinary nutritive and other exciting circumstances of animal life. This is the state of saturation that becomes a subject of important concern, both as to its general safety and to its remedial agency.

All medicines capable of obtaining an ascendancy over the existing actions of vital power, whether healthy or morbid, must exert an energy that deserves to be scrupulously estimated. It is difficult to say, *a priori*, what may be the incidental effects of any medicinal agents that have for their ultimate object a remedial change in the morbid actions of life. Before the system can be sufficiently saturated with the influence proposed to be excited, ailments of various kinds might arise. The visceral functions may be disordered, important secretions perverted, and the different textures inordinately stimulated. These are speculative obstacles, but they may prove practical impediments to seeking from medicinal saturation what might be more safely and effectually obtained from a more limited agency.

The natural state of vital action is provided with too many inherent conditions of safety to admit of being permanently superseded by adventitious influence, whether of the morbific or remedial kind. It is only, therefore, when established disease indicates the necessity of endeavouring to superinduce an altered action in the exertions of living power, that an occasion arises for pushing powerful medicines to the point of saturation. Medicinal efficiency, carried to the extent of affecting the inherent condition of vital action, is a powerful operation, and may induce radical and lasting changes in the organic processes of life. Whatever may be effected by medicinal saturation, it is neither conceivable nor desirable that it should be permanent. It will prevail no longer than until the recruiting and regenerating energy of healthy action shall be enabled to resume its natural and habitual ascendancy.

If health should not speedily supervene the removal of disease by medicinal agency, the remedy may perhaps prove as injurious as the disease itself, by substituting its own influence for that of the healthy state, which would not be a curative exchange. No adequate substitute can be made for the healthful state; nor, indeed, can any change be lasting, that is not closely allied to the condition of health. Diseases are often unyieldingly obstinate, but they are variable, and have not the steady uniformity that characterises the natural and healthy state of vital action. All remedies are designed to control and subdue the force of disease. This object can only be effected by means adapted to that end; and these means will be found in all the various degrees of medicinal agency, from slight local excitement to that of systematic saturation.

The salutary influence of medicinal saturation is exerted in overcoming the diseased actions of vital power, so as to enable the innate disposition to the healthy state to resume its station, and maintain its natural predominance. Medical agency may be remedial in controlling or subduing various morbid states, without requiring to be carried to the extent of saturating or filling the system with its influence to a degree as not to admit of its being carried further, consistently with the safety of life. If medicinal efficiency, whether moderately exerted or pushed to the extreme point of saturation, should be subversive of the disease against which it may be directed, the desired benefit is obtained. When salutary effects can be derived from medicinal operation that would be lenient in its action, it would be preferable to storing the system with its saturating agency. In cases of extreme inveteracy, where temporary benefit may be afforded, but no radical and lasting relief without the utmost efficacy of medicinal power, it becomes necessary to subject the actions of life to its full and unrestrained influence. Not wholly to subdue an existing disease, is to effect no permanent cure, as the slightest remnant of morbid action may progressively augment its force, until it be finally reproduced in all its original violence.

Medicinal agents, therefore, to be curative, must operate complete and durable changes in the diseased action of vital power, or the effect will be no more than temporary relief; an abatement only in the violence of the symptoms, rather than a renewal of the cause on which the morbid appearances depend. There are various diseases arising from a constitutional disposition, and others of slow formation and long existence, that cannot be eradicated without the full efficacy of medicinal saturation. In these cases, the object is to overcome a state of disease that may be threatening to life; it will be therefore requisite to combat such affections by means adequate to the intention to be fulfilled, even though the severity of the remedy may not be wholly devoid of risk. The calculation, under such circumstances, should proceed on a choice of difficulties, and the best should determine the election to be made.

The diseases which originate from an altered or distempered action of vital power, unattended by any disorganised structure, are not necessarily incurable: on the contrary, if the texture in which they more particularly arise should not give way, but remain entire, it would hardly be possible for the ailment to be perpetuated. It is the property of all unnatural actions in the animal economy to wear themselves out. If the healthy stimulus of vital power be not duly applied, disorder will result, which will linger until a more natural excitement shall take place. This may arise from a renovated tone of the affected part, or from a renewal of the precise circumstances that produced the condition of health. If these circumstances do not naturally present, they may perhaps be furnished by medicinal aid. The agents that would contribute to produce the salutary state would supply the necessary remedy; and these must be employed to the extent that may be necessary to effect the beneficial changes that may be required. Whether this influence may be excited by the minimum or maximum doses of medicinal agency, the curative object, if possible, should be accomplished, and the necessary degree of medicinal power should be used for that purpose. Transient impressions of medicinal agency may, in some cases of disease, be sufficient to subdue the altered action on which they may depend, by giving an occasion, during its removal, for a resumption of the healthy state; but, when the morbid action is more established, when the healthy state has been long superseded, and kept in a state of abeyance, it will be necessary to excite the vital power in such a manner as shall effectually superinduce on the diseased action the healthful condition of life. Medicines of much power and efficacy are often required to exert their most energetic influence to subdue this inveteracy of ailment, and to re-instate fully and durably the salutary state of vital action.

What are called the more active or herculean medicines are required in these complicated and difficult cases of disease,—such as the various preparations of quicksilver, the mineral acids, the alkalies, the different kinds of narcotics, particularly belladonna, hyoscyamus, cicuta, opium, and digitalis; the sulphates of zinc and iron, tartarised antimony, arsenic, acetate of lead, bismuth, iodine, hydrocyanic acid, colchicum, elaterium, gamboge, hellebore, squill, &c. These and other powerful medicinal agents may be carried the length of saturating the system, so as not to allow of being extended beyond that limit. Were they to be urged further, either the stomach would reject them in the first instance, or an ulterior excitement would be produced throughout the frame, that would disorder the various functions of life, and induce a state that, if protracted, would be not less decidedly, and perhaps seriously, morbid than that which was intended to be cured. This is the period for desisting from active medicine, that may have been taken to the extent of saturating the system with its properties, lest unwarrantable mischief might result from its continuance.

The morbid influence of indefinite medicinal saturation is such as should awaken much caution in proceeding with active medicine to that length; and, when it has reached it, the utmost vigilance should be observed in restraining and counteracting its hurtful effects. If, instead of diminishing, and ultimately overcoming, the disease for which this extent of remedy was employed, increased ailment should be produced, it will be necessary to discontinue it before any exasperation may have been given to the prevailing malady, and any additional ailment may have been occasioned by the medicinal saturation. It should be remembered that medicinal agency is not salutary, but in as far as it removes disease: if it be exerted either in connexion with disordered action, or in any way that may be inconsistent with the condition of health, it is unnatural, and may in strictness be regarded as producing a morbid effect. Diseases arise from various causes, and noxious agents should be narrowly watched, lest they should escape observation, and imperceptibly and confusedly inflict serious injury. Ailments often become independent of their causes; the morbid effect has resulted from an unhealthful impression on the vital power, which effect will continue after the original impression that produced it may have been removed, or may never cease to operate. The subsequent cause of the morbid state becomes the altered condition, and will not depend for its continuance on the circumstances that may have first occasioned it. Thus, in applying appropriate remedies, the morbid state should be exclusively considered, and a power that may be capable of overcoming it should be directed against it. It will be necessary to graduate and adapt that power to the exigency of the case, so that a consistent and an adequate mode of relief may be afforded. Insufficient agency would be unavailing, whilst excessive efficiency may render the remedy not less noxious than the disease intended to be removed.

The evils resulting from an inordinate, immoderate, and an inapplicable use of medicine, are great and manifold, and should be avoided with the most scrupulous and forbearing care. When it is proposed to alleviate human suffering,—when the earnest request and the anxious desire are to diminish the severity of affliction, it is a wretched accumulation of distress to aggravate the existing malady by the means that may be employed for effecting a cure. When amendment does not arise from the use of any particular medicine, it should be varied in such a manner as may appear to be most likely to accomplish the curative object. To persist in unavailing medicinal agency is, perhaps, to lose the opportunity of relieving, and to endanger further ailment by superadding an injurious, instead of a remedial, influence. No effective medicine can have its use indefinitely prolonged, without producing either salutary or pernicious effects. The actions of life cannot be subjected to strong medicinal efficiency during a long period, either inertly or with impunity: if benefit should not speedily accrue from it, additional disease will be apt to result from such unprofitable excitement.

Mercury, arsenic, steel, cicuta, digitalis, and various other active medicines, by long continuance, so saturate the system with their respective influence, that they cannot be persisted in without immediate inconvenience, and serious eventual mischief. If the powers of life be agitated by unnatural excitement,—if they be held in a state of morbid action by any particular agent, whether it may have been designed to be remedial, or it be of an incidental description, the diseased effect will be nevertheless positive and hurtful, if it be allowed to exert its pernicious agency. The diseased action, that may be respectively denominated mercurial, arsenical, chalybeate, narcotic, &c. is not less important and threatening than that which might arise from any other accidental cause of morbid derangement.

Disorders resulting from an undue employ of medicinal agents are often more violent and unmanageable than those proceeding from less definite and more obscure causes. Before an apprehension may be entertained of the deleterious operation of medicine,—before it may be suspected that its hurtful agency is identifying itself with the action of living power, so as to produce a morbid state, great and complicated mischief may have been incurred, and that, too, in the delusive calculation that a remedial process was working its salutary course.

The insidious and grave nature of this imperceptible injury suggests the propriety of seasonably desisting from the use of powerful medicines, when the desired benefit has not been obtained. A temporary discontinuance of even the best and most certain remedies, when the curative object is not affected in due time, is advisable, as it at once secures against the evils of prolonged saturation, and tends to render the powers of the same medicine afterwards more availing in effecting its salutary purpose. If pernicious saturation should not result from an undue use of medicinal agency, it may, on the other hand, become quite inert, owing to a familiarity that may arise between the qualities of the medicine and the susceptibility in the living power for being affected by them. Early impressions in medicinal agency may be strong, and they may, and often do, continue so, even to the extent of occasioning much ultimate mischief; but they likewise have a tendency progressively to lose of the original force, until at length the effect will altogether cease: thus, the natural and usual aptitude subsisting between the agent and the living power, becomes by habit neutralised, and wholly inefficient.

*Taunton; January 13th,* 1824.

